# Correction: Tantalum(v) 1,3-propanediolate β-diketonate solution as a precursor to sol–gel derived, metal oxide thin films

**DOI:** 10.1039/d0ra90092c

**Published:** 2020-08-28

**Authors:** Christopher Beale, Stefanie Hamacher, Alexey Yakushenko, Oumaima Bensaid, Sabine Willbold, Guillermo Beltramo, Sören Möller, Heinrich Hartmann, Elmar Neumann, Gregor Mussler, Alexander Shkurmanov, Dirk Mayer, Bernhard Wolfrum, Andreas Offenhäusser

**Affiliations:** IBI-3, Bioelectronics, Forschungszentrum Jülich GmbH D-52425 Germany a.offenhaeusser@fz-juelich.de; RWTH Aachen University Templergraben 55 D-52062 Germany; Fraunhofer Research Institute for Microsystems and Solid State Techologies D-80686 Munich Germany; ZEA-3, Analytics, Forschungszentrum Jülich GmbH D-52425 Germany; IBI-2, Mechanobiology, Forschungszentrum Jülich GmbH D-52425 Germany; IEK-1, Materials Synthesis and Processing, Forschungszentrum Jülich GmbH D-52425 Germany; Helmholtz Nano Facility, Forschungszentrum Jülich GmbH D-52425 Germany; PGI-9, Semiconductor Nanoelectronics, Forschungszentrum Jülich GmbH D-52425 Germany; Neuroelectronics, Munich School of Bioengineering, Department of Electrical and Computer Engineering, Technical University of Munich (TUM) D-85748 Garching Germany

## Abstract

Correction for ‘Tantalum(v) 1,3-propanediolate β-diketonate solution as a precursor to sol–gel derived, metal oxide thin films’ by Christopher Beale *et al.*, *RSC Adv.*, 2020, **10**, 13737–13748, DOI: 10.1039/D0RA02558E.

The authors regret that the plasma treatment and printing parameters were reported incorrectly in the subsection “Deposition on a-SiO_2_ for UV/Vis spectrophotometry” in the Experimental section of the original article.

Before printing, the substrate for both samples was subjected to an argon plasma treatment for 5 minutes (150 W, 0.6 mbar). The plasma power is now corrected to be the same as stated in the “Deposition on a-SiO_2_ for Raman/XRD” subsection, where originally it was incorrectly stated that “the power was set slightly higher” for the Raman/XRD samples. For both the acetylacetone and benzoylacetone inks, the inks were printed on their respective substrates with a 75 μm drop pitch having dimensions of 400 × 220 drops to create a uniform layer.

The correct section is as follows:

## Deposition on a-SiO_2_ for UV/Vis spectrophotometry

0.5 mm thick a-SiO_2_ was chosen as the best substrate for transparency in the near UV range. Before printing, the substrate was subjected to an argon plasma treatment for 5 minutes (150 W, 0.6 mbar) using the NANO plasma oven (Diener electronic GmbH + Co. KG, Germany).

Product from the proposed method with acetylacetone was diluted with DEGEE, with the dilution containing 706 mg of DEGEE (70 wt%) and 308 mg of product (30 wt%). The ink was then printed on the substrate with a 75 μm drop pitch having dimensions of 400 × 220 drops to create a uniform layer.

For benzoylacetone, the dilution contained 732 mg DEGEE (69 wt%) and 328 mg of product (31 wt%). The ink was then printed with a drop pitch of 75 μm to create uniform layers (the benzoylacetone ink did not spread as well as the acetylacetone ink), having dimensions of 400 × 220 drops to create a uniform layer.

The Royal Society of Chemistry apologises for these errors and any consequent inconvenience to authors and readers.

## Supplementary Material

